# Lumbosacral Spinal Tuberculosis: Clinical Spectrum, Diagnostic Challenges, and Short-Term Management Outcomes in an Elderly Cohort

**DOI:** 10.7759/cureus.111298

**Published:** 2026-06-22

**Authors:** Yashoverdhan Kag, Anshul Singh, Sandesh Khandelwal, M.K. Jain, Megha Jain

**Affiliations:** 1 General Medicine, LN Medical College and Research Center, Bhopal, IND; 2 Neurology, LN Medical College and Research Center, Bhopal, IND; 3 Neurosurgery, LN Medical College and Research Center, Bhopal, IND; 4 Radiology, LN Medical College and Research Center, Bhopal, IND

**Keywords:** low back pain with neurological deficit, lumbosacral tuberculosis, pathophysiology of spinal tuberculosis, pott’s spine, pott’s spine with neurodeficit

## Abstract

Background: Spinal tuberculosis most commonly affects the thoracic and thoracolumbar spine, whereas lumbosacral involvement is uncommon and may mimic degenerative lumbar spine disorders, often resulting in delayed diagnosis. This study describes the clinical presentation, radiological characteristics, diagnostic challenges, and short-term management outcomes of patients with lumbosacral spinal tuberculosis.

Methods: A retrospective observational study was conducted at a tertiary care center between November 2024 and April 2025. A total of 52 patients with suspected infective spinal pathology presenting with chronic non-traumatic low back pain and/or neurological symptoms were evaluated; 19 were diagnosed with spinal tuberculosis. Six patients with lower lumbar or lumbosacral involvement were included in this case series. Clinical features, laboratory findings, imaging characteristics, treatment strategies, and short-term follow-up outcomes were reviewed.

Results: Of the 19 patients diagnosed with spinal tuberculosis, six (31.6%) demonstrated lumbosacral involvement. Patient age ranged from 47 to 83 years, with five patients aged above 60 years. Clinical presentations included chronic low back pain, radiculopathy, neurogenic claudication, motor weakness, and bowel/bladder dysfunction. MRI demonstrated spondylodiscitis with paradiscal involvement in all cases, while prevertebral or psoas collections were observed in two patients. Five patients underwent surgical decompression and stabilization in addition to antitubercular therapy (ATT), whereas one patient was managed conservatively. All patients demonstrated short-term clinical improvement or stabilization during follow-up.

Conclusions: Lumbosacral spinal tuberculosis may present with diverse clinical manifestations that closely resemble degenerative lumbar spine disorders, creating significant diagnostic challenges. Early MRI evaluation and a high index of clinical suspicion are essential for timely diagnosis and treatment. Awareness of this atypical anatomical pattern is essential for timely, multidisciplinary management. These findings are descriptive and intended to generate hypotheses for future prospective research.

## Introduction

Spinal tuberculosis is recognized as the most clinically significant form of extrapulmonary tuberculosis owing to its capacity to cause severe neurological complications [1]. These complications arise primarily from spinal cord or cauda equina compression resulting from extensive spread of infection into the spinal canal [2]. When the thoracic spine is affected, kyphotic deformities commonly develop, increasing the likelihood of long-term neurological deficits [3]. Early diagnosis and prompt initiation of therapy are therefore essential to prevent residual spinal deformity and irreversible neurological damage [4].

Extrapulmonary tuberculosis, and spinal tuberculosis in particular, continues to present diagnostic challenges because of the limited sensitivity of standard molecular tests such as cartridge-based nucleic acid amplification tests (CBNAAT/GeneXpert) and line probe assays [5]. CBNAAT is currently the preferred first-line molecular investigation for tuberculosis diagnosis, given its higher diagnostic yield compared to acid-fast bacillus (AFB) smear microscopy. In contrast, magnetic resonance imaging (MRI) has demonstrated superior sensitivity (approximately 96%-100%) and specificity (80%-88%) and remains the preferred imaging modality for early detection and evaluation of spinal tuberculosis [5, 6]. Spinal tuberculosis constitutes more than half of all musculoskeletal tuberculosis cases, with the thoracic and thoracolumbar segments being most frequently involved [7].

Lumbosacral spinal tuberculosis is rare, and only a limited number of such cases have been documented in the literature [8]. Clinically, spinal tuberculosis typically presents with chronic back pain, spinal deformities, lower-limb weakness, and, in advanced cases, paraplegia [9]. When the lumbosacral region is affected, however, initial symptoms may closely mimic degenerative conditions such as lumbar disc herniation or spinal canal stenosis, leading to misdiagnosis or diagnostic delays [10]. This is further compounded by the frequent absence of early neurological signs in lumbosacral tuberculosis [10].

This retrospective study evaluates consecutive patients presenting with non-traumatic low back pain associated with neurological symptoms and/or radiological suspicion of infective spinal pathology, assessed across the departments of medicine, neurology, neurosurgery, and orthopedics between November 2024 and April 2025. Of 52 patients screened, 19 were diagnosed with spinal tuberculosis on clinico-radiological grounds, including six cases involving the lumbar/lumbosacral region. The primary objective of this study is to describe the clinical presentation, radiological features, diagnostic challenges, and short-term management outcomes of patients with lumbosacral spinal tuberculosis. Possible pathophysiological mechanisms are discussed in the context of existing literature as hypothesis-generating observations.

## Materials and methods

Study design and setting

This was a retrospective, hospital-based, single-center observational study conducted across the departments of medicine, neurology, neurosurgery, and orthopedics at LN Medical College and Research Center, Bhopal, India, between November 2024 and April 2025. All consecutive patients meeting the predefined screening criteria who presented during the study period were included for evaluation. The study aimed to evaluate the clinical presentation, radiological findings, and short-term management outcomes of patients diagnosed with lumbosacral spinal tuberculosis.

Study population

A total of 52 consecutive patients presenting with chronic non-traumatic low back pain associated with neurological symptoms and/or radiological suspicion of infective spinal pathology were systematically screened during the study period. Among them, 19 patients were diagnosed with spinal tuberculosis based on multimodal clinical, laboratory, and radiological criteria. Of these 19 patients, six demonstrated lower lumbar or lumbosacral involvement and were included in this case series analysis.

Inclusion and exclusion criteria

Inclusion Criteria

The study included patients aged above 18 years presenting with chronic non-traumatic low back pain (duration ≥ 4 weeks) associated with neurological manifestations such as radiculopathy, neurogenic claudication, motor weakness, or bowel/bladder dysfunction, and radiological or clinical suspicion of infective spinal pathology.

Exclusion Criteria

Patients with acute spinal trauma, history of previous spinal instrumentation or surgery, pregnancy, confirmed HIV infection or other immunocompromised status, prolonged steroid or immunomodulator therapy, confirmed malignancy or metastatic disease, severe systemic comorbidities likely to confound outcome assessment, or inability to undergo MRI evaluation.

Mimic pathologies, including pyogenic spondylodiscitis, fungal infections, and metastatic malignancy, were considered and excluded through multidisciplinary clinico-radiological assessment. Features favoring tuberculosis over pyogenic infection included the chronic indolent clinical course, absence of acute sepsis, characteristic MRI findings (paradiscal pattern, psoas/cold abscess), absence of an identifiable pyogenic source, positive interferon-gamma release assay (IGRA) or Mantoux test when performed, and favorable clinical response to antitubercular therapy (ATT).

Diagnostic criteria

The diagnosis of spinal tuberculosis was established using a multimodal clinico-radiological and laboratory-based approach adapted from the diagnostic principles described by Rajasekaran et al. (2018) [3]. Table 1 served as a supportive diagnostic framework. Diagnosis was considered established when at least one parameter from each of three domains: clinical, laboratory, and radiological, was present in the context of overall clinico-radiological correlation and multidisciplinary evaluation, rather than the fulfillment of isolated criteria alone. In surgically managed patients, tissue biopsy findings and CBNAAT/GeneXpert results were additionally sought whenever clinically feasible and available.

**Table 1:** Clinical, laboratory, and radiological (MRI) features of lumbosacral spinal tuberculosis

Clinical	Laboratory	Radiological (MRI)
(a) Subacute to chronic disabling low back pain	(a) Elevated erythrocyte sedimentation rate (ESR) and C-reactive protein (CRP)	(a) Loss of normal marrow signal intensity and edema and bony erosion,
(b) Spinal deformity like kyphosis, cold abscess, Gibbus deformity	(b) Interferon-gamma release assay (IGRA)	(b) T1-weighted: hypointense signals on bone
(c) Neurological deficits (e.g., paraparesis, bladder/bowel dysfunction)	(c) Mantoux test	(c) T2/short tau inversion recovery (STIR): hyperintense signals (inflammation or abscess)
(d) Constitutional symptoms may be absent or mild	(d) Chest X-ray (CXR), abdominal ultrasound (USG) for systemic tuberculosis evaluation	(d) Spondylodiscitis with heterogeneous enhancement on contrast

As this was a retrospective study, microbiological and histopathological confirmation (including *Mycobacterium tuberculosis* (MTB) culture, CBNAAT, AFB smear, and histopathology) was not uniformly available in all patients. Sputum or bronchoalveolar lavage (BAL) nucleic acid amplification testing (NAAT) was not routinely performed in the absence of respiratory symptoms. Where available, these results have been incorporated into the diagnostic assessment for individual cases. 

ATT was administered in accordance with prevailing National Tuberculosis Elimination Programme (NTEP) recommendations for drug-susceptible extrapulmonary tuberculosis, using the standard first-line four-drug regimen (HRZE: isoniazid, rifampicin, pyrazinamide, and ethambutol) during the intensive phase, followed by continuation-phase therapy [11]. One patient developed ATT-induced hepatitis, most likely attributable to pyrazinamide, and the regimen was modified according to institutional practice and NTEP-guided principles for ATT-induced liver injury. It should be noted that, unlike pulmonary tuberculosis, there are no specific NTEP guidelines exclusively dedicated to spinal tuberculosis; management therefore relies on clinical judgment, disease severity, neurological involvement, and multidisciplinary decision-making.

Surgical indications

Five patients underwent surgery based on established indications, including progressive neurological deficit, mechanical spinal instability, and failure of or inadequate response to conservative management. One patient (Case 2) was managed conservatively with ATT alone, given the absence of motor deficit and satisfactory clinical response.

Follow-up and outcome assessment

Patients were followed up at one month and three months following initiation of treatment. Outcome assessment was based on clinical parameters, including pain severity, neurological recovery (motor power grading), ambulation status, and bowel/bladder function. As this was a retrospective study, standardized outcome measures such as the Oswestry Disability Index (ODI), Visual Analog Scale (VAS) for pain, or Frankel/Japanese Orthopaedic Association (JOA) neurological scores were not uniformly available and were therefore not applied. Outcomes reported represent short-term clinical improvement and stabilization rather than definitive long-term treatment success.

Ethics statement

This retrospective observational study involving human participants was conducted in accordance with the ethical principles of the Declaration of Helsinki. Institutional Ethics Committee approval (or waiver for retrospective case series review) was obtained prior to data collection. Patient confidentiality was maintained throughout the study.

## Results

Demographic and clinical overview

Among 52 consecutive patients evaluated for suspected infective spinal pathology during the study period, 19 were diagnosed with spinal tuberculosis, including six patients with lower lumbar/lumbosacral involvement. Patient ages ranged from 47 to 83 years, with the majority (five of six patients) aged above 60 years. Chronic low back pain and radiculopathy were the most common presenting complaints. Three patients had associated motor weakness and bowel/bladder involvement. MRI demonstrated paradiscal involvement in all six patients; prevertebral or paravertebral soft tissue collections or psoas abscesses were observed in two patients, consistent with previously described imaging findings in spinal tuberculosis [12]. Elevated ESR (reference range: 0-20 mm/hr in males; 0-30 mm/hr in females) and CRP (reference range: <6 mg/L) were noted in five patients; one patient had normal inflammatory markers but a positive IGRA. Five patients underwent surgical decompression and stabilization in addition to ATT; one patient improved with conservative management alone. Multilevel vertebral involvement (L4-S1) was observed in two patients. All six patients demonstrated clinical improvement at follow-up, with no evidence of disease progression at three months. A summary of all six cases, including the diagnostic basis for each, is provided in Table 2.

**Table 2:** Summary of clinical, radiological, and therapeutic profiles of six cases of lumbosacral spinal tuberculosis All cases were diagnosed on clinico-radiological grounds; microbiological or histopathological confirmation was sought intraoperatively in surgically managed patients, but was not uniformly available retrospectively. ATT: antitubercular therapy; CBNAAT: cartridge-based nucleic acid amplification test; CRP: C-reactive protein (reference range: <6 mg/L); ESR: erythrocyte sedimentation rate (reference range: 0–15 mm/hr males); HRCT: high-resolution computed tomography; HRZE: isoniazid, rifampicin, pyrazinamide, and ethambutol; IGRA: interferon-gamma release assay; TB: tuberculosis; TLC: total leukocyte count.

Case/Age/Sex	Presenting Deficit	Symptom Duration	Key MRI Findings	Intervention	Early Outcome	One-Month Follow-Up	Three-Month Follow-Up	Diagnostic Basis
1/ 73 years/M	Bilateral proximal lower limb weakness; bowel and bladder dysfunction	Low back pain: eight months; motor deficit: two months	L5–S1 vertebral destruction with disc involvement; epidural extension; canal compromise	L5 laminectomy + discectomy + ilio-lumbar screw fixation; ATT (HRZE regimen)	Symptomatic improvement; pain reduced	Standing with support; improving bowel continence	Independent ambulation; near-normal bladder function	Elevated ESR/TLC; HRCT: right upper lobe cavity (pulmonary TB); MRI: spondylodiscitis L5–S1; intraoperative tissue: CBNAAT + histopathology
2/ 83 years/M	Left lower limb radiculopathy; no motor deficit	Four months	Grade I–II anterolisthesis L4–L5; degenerative changes L2–L5; left psoas cold abscess	Conservative: ATT (HRZE) + supportive care	Significant pain relief by 4 weeks	Marked reduction in radicular pain; improved mobility	No radiological progression; ESR/CRP normalised	Normal ESR/TLC; positive IGRA; HRCT: normal; MRI: psoas collection; clinical response to ATT confirmed
3/47 years/F	Neurogenic claudication (~200 m); no motor deficit	Three months	L5–S1 discitis; mild–moderate spinal canal stenosis	L4–L5 laminectomy + discectomy + fusion + fixation; ATT (HRZE)	Improved walking tolerance	Claudication significantly reduced; no new neurological signs	Symptom-free; MRI stable	Elevated ESR; MRI: active L5–S1 discitis; response to ATT confirmed
4/61 years/F	Right lower limb radiculopathy; no motor deficit	Three months	T2 hyperintense collection L4–L5 disc; vertebral body destruction; no epidural compromise	L3–L4 laminectomy + L2–L5 fusion and fixation; ATT (HRZE)	Mild residual pain; stable neurologically	Ambulating with mild discomfort; improving	No disease progression; on maintenance ATT	Elevated ESR and CRP; Mantoux and IGRA negative; MRI: L4–L5 spondylodiscitis; ATT response confirmed
5/68 years/F	Left lower limb weakness (MRC 4−/5); bilateral radiculopathy	Two months	L4–S1 multilevel vertebral destruction; disc narrowing; prevertebral and epidural collections	L5 laminectomy + L3–S1 pedicle screw fixation; ATT modified after ATT-induced hepatitis	Motor improvement; hepatic recovery on modified ATT	Hepatic enzymes normalised; tolerating modified regimen	Lower limb strength improving; residual mild pain	Elevated ESR and CRP; MRI: multilevel L4–S1 spondylodiscitis; intraoperative specimen: CBNAAT + histopathology; ATT response on modified regimen confirmed
6/65 years/M	Left lower limb weakness; bilateral radiculopathy; bowel and bladder dysfunction	Two months	T2 hyperintense L4–L5 disc collection; vertebral destruction; epidural extension	L2–L5 laminectomy + microdiscectomy + fusion + fixation; ATT (HRZE) + short corticosteroid course	Marked symptomatic improvement	Improving bladder control; ambulant with support	Mild residual pain; neurologically stable; no progression	Elevated ESR and CRP; MRI: L4–L5 spondylodiscitis with epidural component; ATT response confirmed

Case series

Case 1

**Figure 1:**
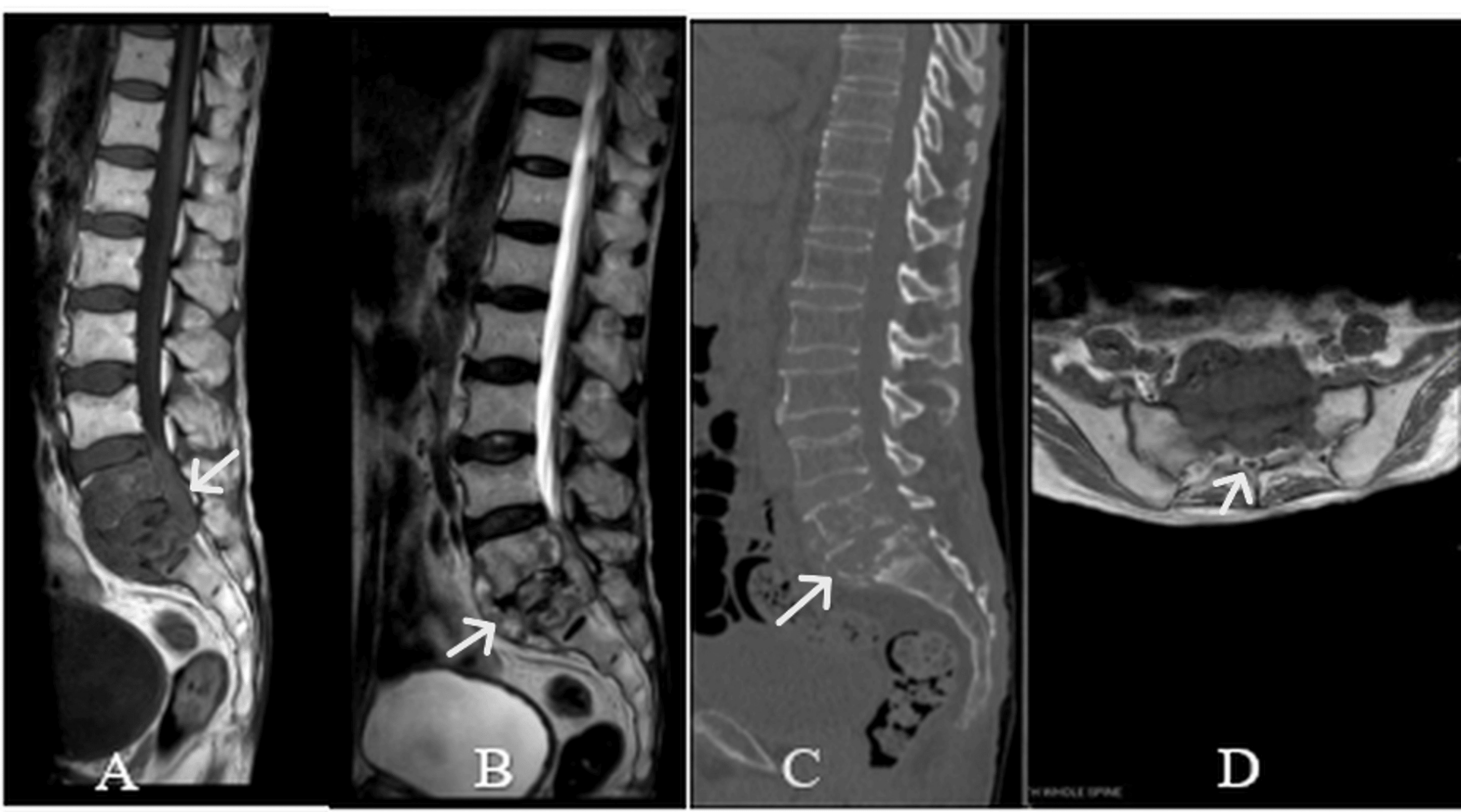
MRI of the lumbosacral spine (Case 1) (A) Sagittal T1-weighted image: hypointense marrow signal at L5 and S1 vertebral bodies (arrow indicates areas of signal loss). (B) Sagittal T2-weighted image: hyperintense inflammatory changes with L5–S1 disc involvement (arrow). (C) Post-contrast sagittal image: heterogeneous enhancement around affected vertebral endplates and intervertebral disc (arrow). (D) Axial T1-weighted image: epidural extension with canal compromise at L5–S1 (arrow). Findings are consistent with infective spondylodiscitis at the lumbosacral junction.

A 73-year-old male patient presented with eight months of progressive low back pain and two months of bilateral proximal lower limb weakness, worsened by lying down, with associated bowel/bladder dysfunction, low-grade fever, and weight loss. Examination revealed localized lumbosacral tenderness and proximal muscle weakness (4/5). Laboratory investigations showed elevated total leucocyte count (total leukocyte count (TLC): 11,700/mm³; reference range: 4,000-11,000/mm³) and elevated ESR (40 mm/hr; reference range: 0-20 mm/hr for males). MRI of the lumbosacral spine demonstrated destruction of the L5 and S1 vertebral bodies with disc involvement, consistent with infective spondylodiscitis (Figure 1). High-resolution computed tomography (HRCT) of the chest revealed a thick-walled cavity with tree-in-bud nodules in the right upper lobe, consistent with pulmonary tuberculosis. The patient underwent L5 laminectomy, discectomy, and ilio-lumbar screw fixation. Standard ATT (HRZE regimen per NTEP guidelines) was initiated. At the three-month follow-up, the patient showed significant symptomatic improvement and regained independent ambulation.

Case 2

**Figure 2:**
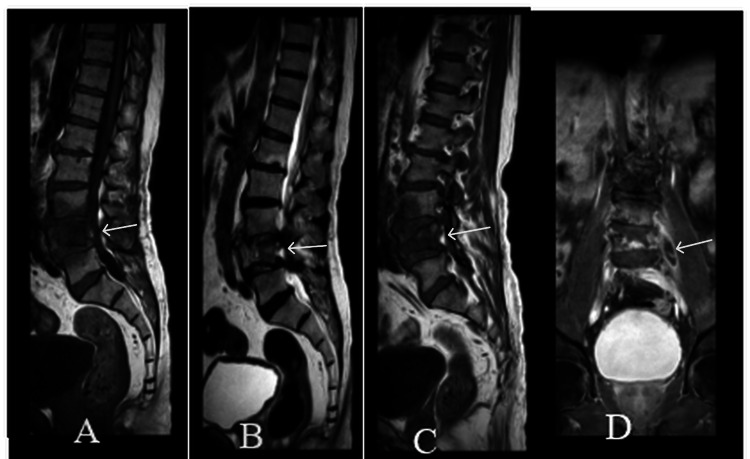
MRI of the lumbosacral spine (Case 2) A) Sagittal T1-weighted image: altered marrow signal at L4 and L5 vertebral endplates (arrow). (B) Sagittal T2-weighted image: hyperintense inflammatory changes with disc involvement at L4–L5 (arrow). (C) Coronal image: left psoas muscle collection consistent with abscess formation (arrow). (D) Axial image: prevertebral soft tissue extension adjacent to the involved vertebral bodies (arrow).

An 83-year-old hypertensive male patient with a prior history of coronary artery bypass grafting (CABG, 2010) presented with four months of progressive low back pain radiating to the left lower limb, aggravated by walking and squatting. Neurological and systemic examinations were unremarkable. Laboratory tests showed normal TLC (9,600/mm³; reference range: 4,000-11,000/mm³) and normal ESR (9 mm/hr; reference range: 0-20 mm/hr for males). MRI revealed grade I-II anterolisthesis of L4 over L5, degenerative disc changes from L2 to L5, and a left psoas-plane prevertebral soft tissue component consistent with a chronic cold abscess (Figure 2). HRCT chest was normal; IGRA was positive. Pyogenic spondylodiscitis was considered but excluded on the basis of the chronic indolent clinical course, absence of acute sepsis, characteristic MRI cold abscess morphology, absence of an identifiable pyogenic source, and positive IGRA. Spinal tuberculosis was diagnosed on clinico-radiological grounds, and ATT with supportive care was initiated. The patient reported significant pain relief within one month with no evidence of disease progression at the three-month review.

Case 3

**Figure 3:**
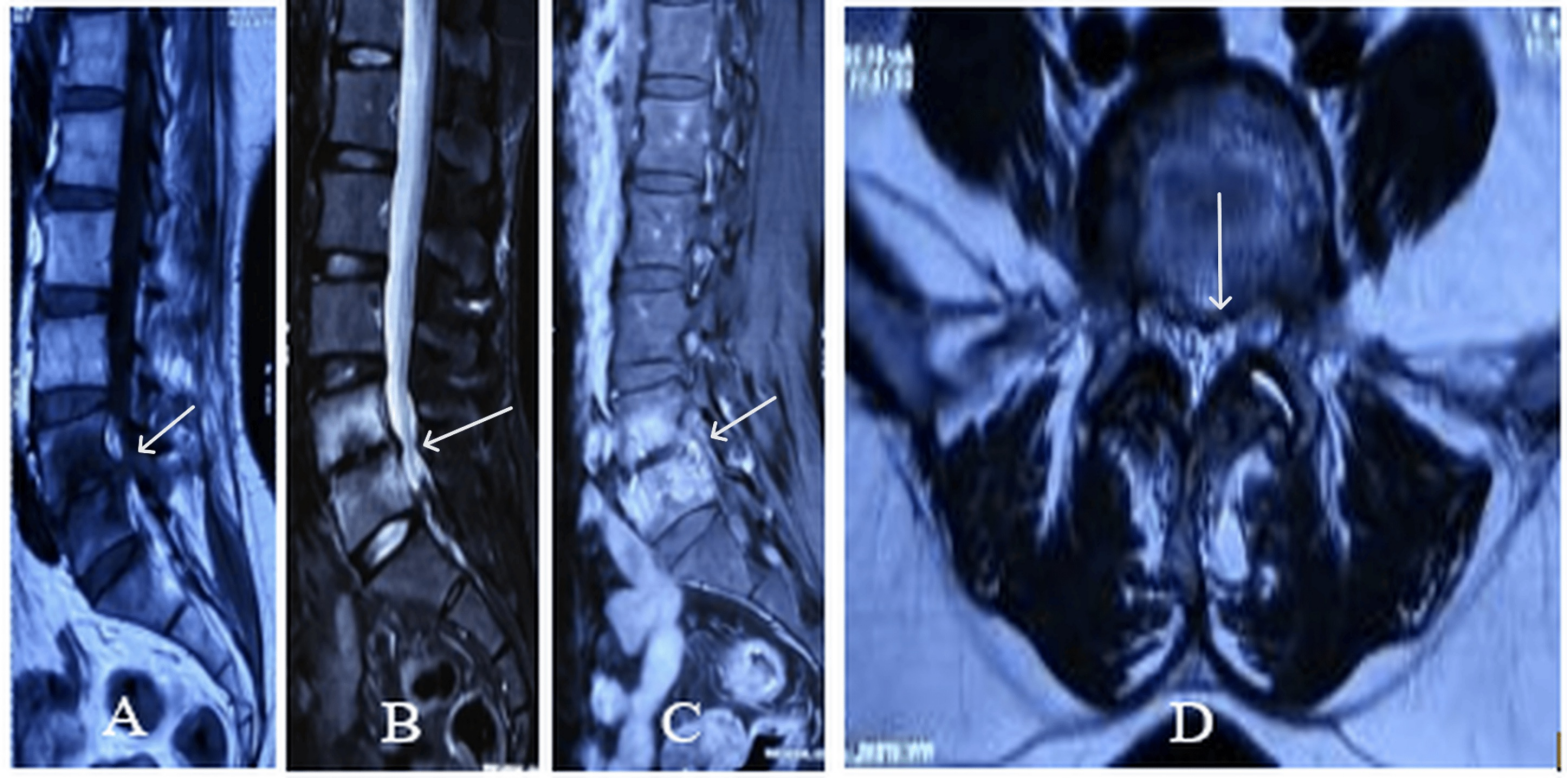
MRI of the lumbosacral spine (Case 3) (A) Sagittal T1-weighted image: hypointense marrow signal at peridiscal regions of L5 and S1 (arrow). (B) Sagittal T2/short tau inversion recovery (STIR) image: hyperintense inflammatory signal with discitis at L5–S1 (arrow). (C) Bone window image: vertebral endplate irregularity and bony destruction (arrow). (D) Axial image: diffuse posterior disc protrusion causing mild thecal sac compression with bilateral nerve root involvement (arrow). Findings are consistent with active infective spondylodiscitis at the lumbosacral junction.

A 47-year-old female patient presented with chronic disabling low back pain and claudication limiting walking to approximately 200 meters, without motor weakness or systemic symptoms. There was no history of trauma, fever, or weight loss. Neurological examination was normal. Laboratory tests showed normal TLC (9,600/mm³; reference range: 4,000-11,000/mm³) and mildly elevated ESR (37 mm/hr; reference range: 0-30 mm/hr for females). MRI revealed L5-S1 discitis with mild-to-moderate spinal canal stenosis (Figure 3). A diagnosis of clinico-radiologically presumed lumbosacral spinal tuberculosis (Pott's disease) was made on an overall clinico-radiological correlation after exclusion of degenerative and pyogenic etiologies. The patient underwent L4-L5 laminectomy with discectomy, fusion, and fixation, along with ATT. She showed symptomatic improvement with no further progression at the three-month follow-up.

Case 4

**Figure 4:**
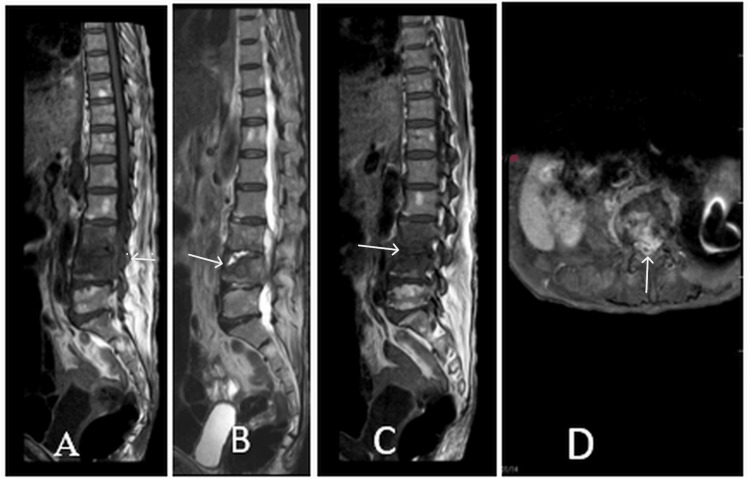
MRI of the lumbosacral spine (Case 4) (A) Sagittal T1-weighted image: hypointense marrow signal changes at L4 and L5 vertebral bodies (arrows). (B) Sagittal T2-weighted image: hyperintense collections within the L4–L5 intervertebral disc space (arrows). (C) Bone window image: vertebral endplate destruction and disc space narrowing (arrow). (D) Axial image: mild spinal canal compromise at the affected level (arrows). Findings are consistent with infective spondylodiscitis at L4–L5.

A 61-year-old female patient presented with a three-month history of progressively worsening low back pain radiating to the right lower limb, aggravated by movement and interfering with daily activities. There was no history of trauma, fever, or weight loss. Examination revealed tenderness over the lumbosacral spine and mild restriction of spinal mobility; neurological evaluation showed no motor or sensory deficits, with preserved reflexes. Laboratory findings demonstrated elevated ESR (45 mm/hr; reference range: 0-30 mm/hr for females) and elevated CRP (18 mg/L; reference range: <6 mg/L). MRI demonstrated T2 hyperintense collections in the L4-L5 intervertebral disc space with adjacent vertebral body destruction, consistent with infective spondylodiscitis (Figure 4). Mantoux and IGRA tests were negative. A clinical diagnosis of presumed Pott's spine was established on the basis of MRI findings, elevated inflammatory markers, symptomatology, and exclusion of pyogenic infection and malignancy. Surgical intervention was indicated, given severe, persistent radicular pain with vertebral destruction and evidence of radiological instability despite conservative treatment. The patient underwent L3-L4 laminectomy with fusion and fixation from L2 to L5, followed by standard ATT and supportive care. Intraoperative specimens were sent for histopathological examination. At the three-month follow-up, only mild residual pain was reported, with no signs of disease progression.

Case 5

**Figure 5:**
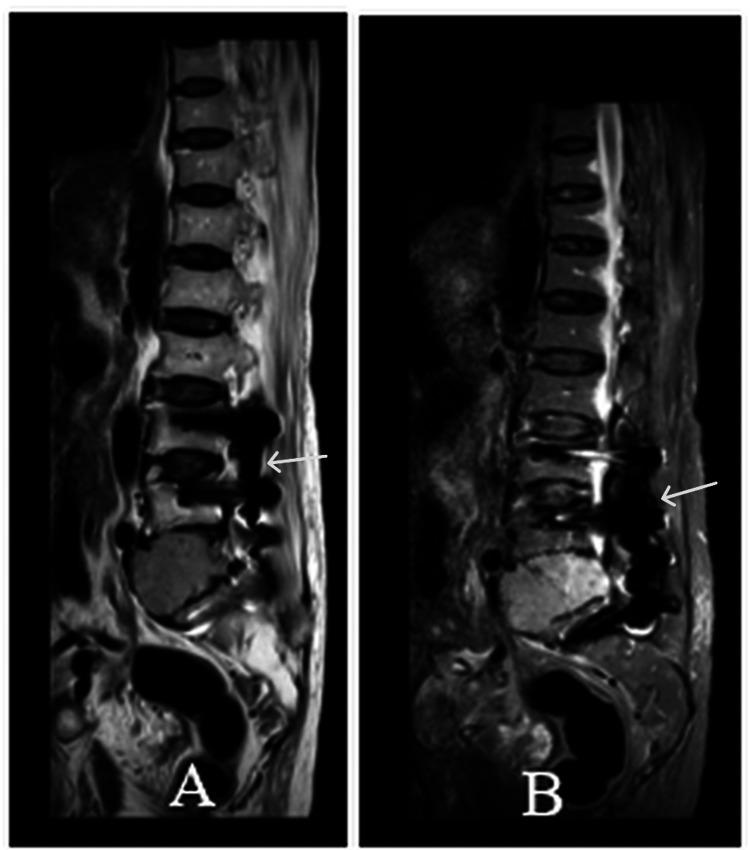
Postoperative MRI (Case 5) (A) Sagittal T2-weighted image: L5 laminectomy changes and posterior spinal fixation extending from L3 to S1 (arrow). (B) Short tau inversion recovery (STIR) sequence: maintained postoperative spinal alignment with reduction in inflammatory soft tissue changes at the operative site (arrow). Note: This figure is presented as a postoperative MRI demonstrating spinal alignment and instrumentation following surgical stabilization. As the corresponding preoperative MRI images were not available for review, direct radiological comparison was not possible. In addition, interpretation may be influenced by metallic susceptibility artifacts related to pedicle screw fixation. Treatment response is described primarily on the basis of clinical improvement and follow-up findings.

A 68-year-old female patient presented with a two-month history of progressive low back pain radiating bilaterally to both lower limbs, along with left lower limb weakness resulting in reduced mobility. Examination revealed lumbosacral tenderness, mild kyphosis, and decreased motor power (4−/5) in the left lower limb; the right limb was neurologically intact. Bowel and bladder functions were preserved. Laboratory investigations showed elevated ESR (52 mm/hr; reference range: 0-30 mm/hr for females) and elevated CRP (22 mg/L; reference range: <6 mg/L). MRI demonstrated L4-S1 vertebral destruction, disc space narrowing, and prevertebral and epidural soft tissue collections, confirming infective spondylodiscitis consistent with spinal tuberculosis (Figure 5). The patient underwent L5 laminectomy with posterior stabilization using pedicle screw fixation from L3-L4 to L4-S1. Postoperatively, standard ATT (HRZE regimen) and structured rehabilitation were initiated. The patient developed ATT-induced hepatitis, most likely related to pyrazinamide; the regimen was modified according to institutional practice and NTEP-guided principles for ATT-induced liver injury. At the one-month follow-up, hepatic function had normalized, and the patient was tolerating the modified regimen well, with improved lower limb strength.

Case 6

**Figure 6:**
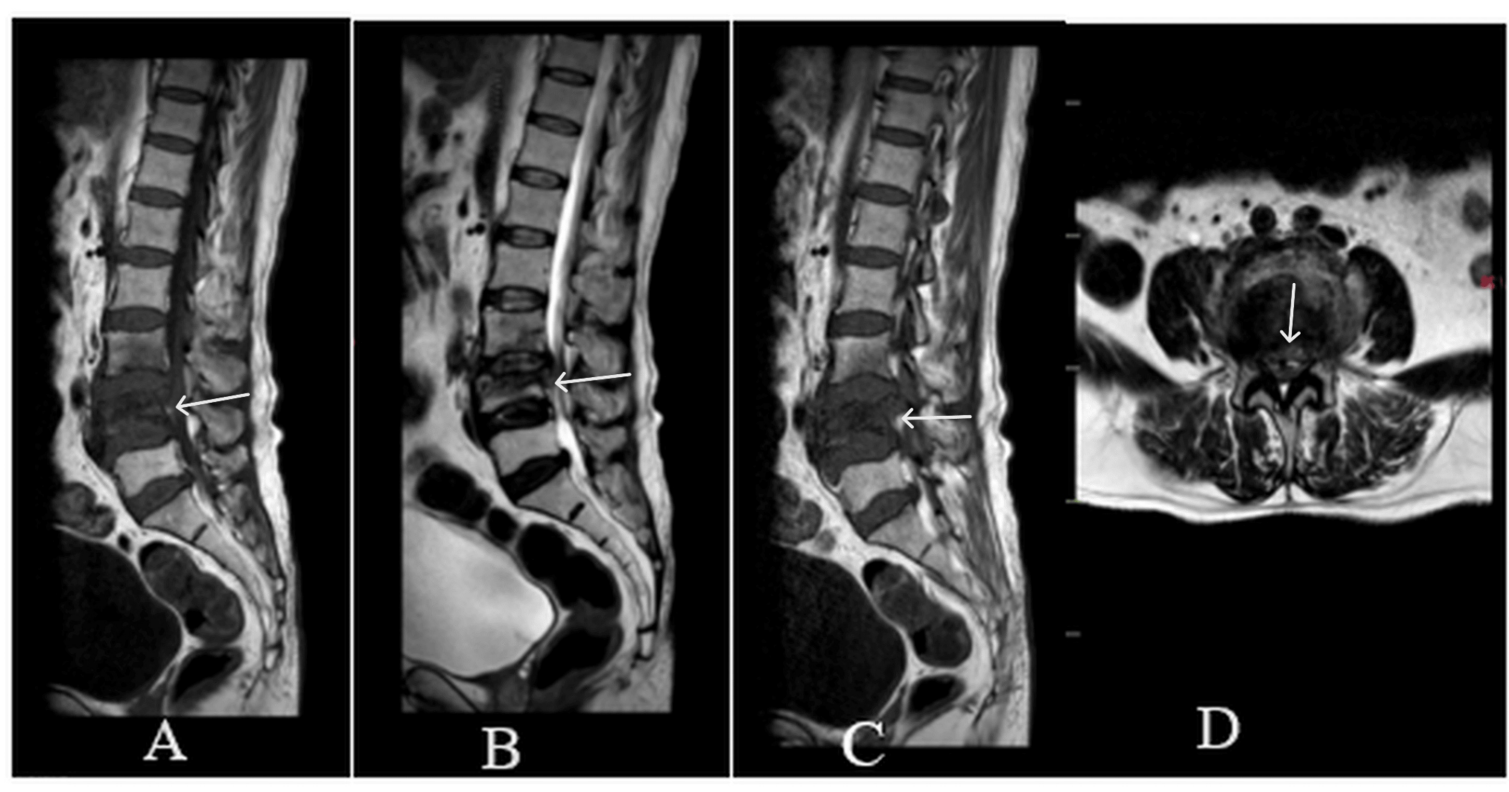
MRI of the lumbosacral spine (Case 6) (A) Sagittal T1-weighted image: hypointense marrow signal at L4 and L5 vertebral bodies (arrow). (B) Sagittal T2-weighted image: hyperintense inflammatory collections within the L4–L5 disc space (arrow). (C) Bone window image: adjacent vertebral destruction and endplate irregularity (arrow). (D) Axial image: epidural extension with mild canal compromise (arrow). Findings are consistent with infective spondylodiscitis at L4–L5.

A 65-year-old male patient with diabetes mellitus and a prior stroke presented with two months of progressive, disabling low back pain radiating to both lower limbs, along with left lower limb weakness and bowel/bladder involvement. No constitutional symptoms (fever, weight loss, or night sweats) were reported. Neurological deficits were more prominent on the left side. Laboratory investigations revealed elevated ESR (48 mm/hr; reference range: 0-20 mm/hr for males) and elevated CRP (16 mg/L; reference range: <6 mg/L). MRI of the lumbosacral spine revealed T2 hyperintense disc collections at L4-L5 with adjacent vertebral body destruction and epidural extension (Figure 6), consistent with spinal tuberculosis (Pott's spine). Surgical decompression with L2-L5 laminectomy, microdiscectomy, and spinal fusion with fixation was performed, given progressive paraparesis with bowel/bladder involvement. Intraoperative tissue was submitted for CBNAAT/GeneXpert and histopathological examination. Standard ATT, along with a short course of corticosteroids, was initiated postoperatively. At the three-month follow-up, the patient demonstrated marked symptomatic improvement, with only mild residual pain and no clinical evidence of disease progression.

## Discussion

Overview of lumbosacral spinal tuberculosis

Lumbosacral spinal tuberculosis, although less frequently reported than thoracic involvement, presents significant clinical and diagnostic challenges owing to the anatomical and biomechanical uniqueness of this region [7]. The lumbosacral junction bears the highest axial load of the vertebral column, serves as a transitional zone for spinal movement, and is in close anatomical relationship with pelvic organs. These features collectively contribute to a distinct pattern of disease presentation, progression, and complication profile that sets lumbosacral tuberculosis apart from classical thoracic or thoracolumbar disease. The following discussion is based on descriptive observations from this small retrospective series and existing literature; mechanistic conclusions should be regarded as hypothesis-generating.

Clinical presentation: atypical yet progressive

Patients with lumbosacral tuberculosis predominantly present with insidious, chronic low back pain [10]. Unlike thoracic tuberculosis, which often manifests with visible kyphosis and prominent constitutional symptoms, lumbosacral tuberculosis may initially mimic degenerative disc disease or mechanical back pain, leading to diagnostic delays [10]. In the present series, all six patients presented with chronic localized back pain; however, only two had constitutional symptoms such as low-grade fever or weight loss. Neurological involvement was observed in five of six patients, ranging from isolated radiculopathy to significant motor weakness and bowel/bladder dysfunction. This pattern is consistent with existing literature suggesting that cauda equina compression, early segmental instability, and root irritation are more prevalent in lumbosacral tuberculosis, attributable to the proximity of neural structures and increased mechanical stress [7]. One patient also presented with neurogenic claudication, an uncommon but noteworthy presentation of tubercular spondylitis [10].

Cold abscess formation in the psoas or paraspinal regions was observed in two cases, with an absence of overt systemic signs of inflammation, reaffirming the characteristically indolent nature of tubercular abscesses [13]. Mild kyphotic changes and focal spinal deformity were noted in patients with multilevel vertebral involvement (L4-S1), highlighting the importance of early structural assessment [12].

Pathophysiological basis for lumbosacral involvement: hypothesis-generating considerations

Multiple factors have been proposed in the literature to predispose the lumbosacral region to tuberculosis infection in selected individuals. The region bears the highest axial mechanical load and is prone to microtrauma, thereby potentially facilitating bacillary seeding [7]. The valveless Batson's venous plexus enables retrograde mycobacterial spread from genitourinary or gastrointestinal tuberculosis foci to the lumbosacral vertebrae [14]. Reduced lymphatic drainage in this region may also permit persistence of infection once established [12]. These mechanisms are discussed here as hypothesis-generating considerations based on previously published literature and are not primary findings of the present retrospective case series.

Radiological and laboratory diagnosis

MRI remains the gold standard for diagnosing spinal tuberculosis owing to its superior soft tissue resolution and ability to detect early marrow edema, epidural extension, and abscess formation, with reported sensitivity of approximately 96%-100% and specificity of 80%-88% [15]. All patients in this series exhibited the paradiscal pattern of involvement, the most common radiological form, manifesting as vertebral body destruction and disc space narrowing. Two patients had associated psoas abscesses and prevertebral soft tissue involvement [12]. Multilevel involvement was observed in two patients, both of whom required surgical stabilization, reflecting the propensity of lumbosacral tuberculosis to cause early segmental instability.

Laboratory findings supported the diagnosis in five of six patients, with elevated ESR and CRP. One patient had normal inflammatory markers but tested positive on IGRA, underscoring the limited sensitivity of serological parameters alone in extrapulmonary tuberculosis [10]. CBNAAT/GeneXpert is currently the preferred first-line molecular diagnostic test, given its higher sensitivity over AFB smear microscopy. Owing to the retrospective nature of this study, CBNAAT and mycobacterial culture results were not uniformly available; this represents a significant limitation and is discussed further below.

Management and short-term outcomes

Five of six patients required surgical decompression and stabilization due to significant neural compression, mechanical instability, or failure of conservative therapy [16,17]. Surgical interventions comprised laminectomy, discectomy, and instrumented fusion [18]. All patients received ATT (HRZE standard first-line regimen per NTEP guidelines [11]) along with a short course of corticosteroids and supportive care, with gradual short-term symptomatic improvement in all cases [16]. One patient developed ATT-induced hepatitis, likely attributable to pyrazinamide, necessitating temporary modification of the regimen according to NTEP-guided principles for ATT-induced liver injury; the regimen was subsequently reintroduced successfully. At short-term follow-up (one and three months), no patient showed evidence of disease progression; however, long-term outcomes were not assessable within the study period.

Clinical implications

This retrospective case series is among the few describing the clinical and radiological features of lumbosacral spinal tuberculosis in a predominantly adult, predominantly older population from a single tertiary center in central India. Unlike classical thoracic spine tuberculosis, which typically presents with pronounced kyphosis and early neurological compromise, lumbosacral tuberculosis manifests subtly and frequently mimics degenerative spinal disorders, resulting in diagnostic delay. The present series underscores the necessity for heightened clinical awareness and early MRI evaluation in patients presenting with atypical lower back pain, to ensure timely initiation of therapy and prevention of neurological deterioration. Findings should be interpreted within the limitations described below and are intended to be descriptive and hypothesis-generating.

Limitations

This study has several important limitations that must be explicitly acknowledged. First, the sample size (lumbosacral TB) was small (n=6), reflecting both the rarity of lumbosacral spinal tuberculosis and the short study duration (six months); findings should therefore be interpreted with caution and not generalized broadly. Second, microbiological and histopathological confirmation was not uniformly available in all patients owing to the retrospective nature of the study; this constitutes a major limitation, as pyogenic spondylodiscitis, fungal infections, and metastatic malignancy can closely mimic the radiological appearance of spinal tuberculosis. Although microbiological sampling should ideally be attempted in all accessible lesions (including cold abscesses), this was not uniformly feasible retrospectively. Consequently, cases are described as clinico-radiologically diagnosed (presumed) spinal tuberculosis. Third, CBNAAT/GeneXpert testing, sputum/BAL NAAT, and mycobacterial culture were not performed or were unavailable in several patients. Fourth, standardized outcome measures (ODI, VAS pain scores, Frankel or JOA neurological grades) were not uniformly recorded, limiting objective outcome comparison. Fifth, the follow-up period was limited to three months, which is insufficient to assess long-term functional and radiological outcomes in a disease requiring nine to 12 months of ATT. Sixth, the cohort was not exclusively elderly; one patient was 47 years old, precluding firm conclusions specific to a geriatric population. Seventh, the absence of standardized epidemiological data on lumbosacral tuberculosis in India and the lack of uniform diagnostic criteria across centers restrict broader generalization. Future prospective multicenter studies with microbiological confirmation, standardized outcome tools, and longer follow-up are warranted.

## Conclusions

Lumbosacral spinal tuberculosis represents a rare yet clinically significant subset of Pott's disease, often presenting with non-specific symptoms that closely mimic degenerative spinal disorders. Its atypical anatomical location, high mechanical stress at the lumbosacral junction, and the delayed onset of neurological signs contribute to frequent diagnostic delays. This small single-center retrospective case series describes the distinct clinical and radiological features of clinico-radiologically diagnosed lumbosacral tuberculosis and emphasizes the critical role of MRI in early detection. Timely recognition is essential to prevent irreversible neurological deficits and mechanical instability. Greater clinical awareness and multidisciplinary evaluation are imperative to differentiate lumbosacral tuberculosis from common degenerative lumbar pathologies and to ensure prompt, targeted treatment. These findings are descriptive and hypothesis-generating; larger prospective studies with uniform microbiological confirmation and longer follow-up are needed to validate these observations and establish evidence-based management guidelines.
